# Nanoscale Vacancy-Mediated Aggregation, Dissociation, and Splitting of Nitrogen Centers in Natural Diamond Excited by Visible-Range Femtosecond Laser Pulses

**DOI:** 10.3390/nano13020258

**Published:** 2023-01-07

**Authors:** Sergey Kudryashov, Galina Kriulina, Pavel Danilov, Evgeny Kuzmin, Alexey Kirichenko, Nikolay Rodionov, Roman Khmelnitskii, Jiajun Chen, Elena Rimskaya, Vladimir Shur

**Affiliations:** 1 Lebedev Physical Institute, 119991 Moscow, Russia; 2 Geology Faculty, Lomonosov Moscow State University, 119899 Moscow, Russia; 3 Institution “Project Center ITER”, 123182 Moscow, Russia; 4 School of Natural Sciences and Mathematics, Ural Federal University, 620000 Ekaterinburg, Russia

**Keywords:** natural IaA+B diamond, highly and lowly aggregated nitrogen centers, femtosecond laser pulses, interstitial vacancy photogeneration, vacancy attachment, photodissociation, vacancy-driven splitting

## Abstract

Natural IaA+B diamonds were exposed in their bulk by multiple 0.3 ps, 515 nm laser pulses focused by a 0.25 NA micro-objective, producing in the prefocal region (depth of 20–50 μm) a bulk array of photoluminescent nanostructured microtracks at variable laser exposures and pulse energies. These micromarks were characterized at room (25°) and liquid nitrogen cooling (−120 °C) temperatures through stationary 3D scanning confocal photoluminescence (PL) microspectroscopy at 405 and 532 nm excitation wavelengths. The acquired PL spectra exhibit a linearly increasing pulse-energy-dependent yield in the range of 575 to 750 nm (NV^0^, NV^−^ centers) at the expense of the simultaneous reductions in the blue–green (450–570 nm; N3a, H4, and H3 centers) and near-IR (741 nm; V^0^ center) PL yield. A detailed analysis indicates a low-energy rise in PL intensity for B2-related N3a, H4, and H3 centers, while at higher, above-threshold pulse energies it decreases for the H4, H3, and N3a centers, converting into NV centers, with the laser exposure effect demonstrating the same trend. The intrinsic and (especially) photo-generated vacancies were considered to drive their attachment as separate species to nitrogen centers at lower vacancy concentrations, while at high vacancy concentrations the concerted splitting of highly aggregated nitrogen centers by the surrounding vacancies could take place in favor of resulting NV centers.

## 1. Introduction

Modern ultrashort pulse (femto- or picosecond) lasers provide almost unlimited opportunities for diverse structural modifications (multi-photon polymerization, densification, void formation, crystallization, etc.) in transparent media via predetermined, well-controlled laser energy deposition inside the focal waist in their bulk [1]. In diamond materials these ultrafast laser writing technologies were harnessed for the inscription of single photoluminescent NV centers in synthetic electronic-grade low-nitrogen diamonds [2], or for marking high-nitrogen natural diamonds via the bulk inscription of photoluminescent QR code arrays [3,4].

Despite these remarkable nanophotonic advances, the underlying microscopic structural paths remain hidden, effectively for two main reasons: The first issue is that, usually, the exact initial structural state of nitrogen impurity is not known once different lowly and highly aggregated nitrogen centers appear in either IR, Raman, optical, or ESR spectroscopy [5], disabling their quantitative characterization as the main precursors for laser-induced solid-state reactions. The obvious second issue is then the photochemical laser effect on either a carbon lattice (the photogeneration of an “interstitial vacancy”, I–V Frenkel pairs [6,7]) or directly on nitrogen centers to induce their dissociation or other activation processes [8]. More generally, strong electronic, anisotropic, or isotropic (high-pressure) stress and temperature perturbations proceed consequently in the laser waist [9], bringing some history to laser-induced transformations of nitrogen centers. Under these circumstances, both the detailed initial characterization of the present lowly and highly aggregated nitrogen centers in addition to their comprehensive post-irradiation 3D scanning photoluminescence spectral analysis become of key importance [10,11,12] once other FT-IR or optical–microspectroscopic tools are either not available or sensitive enough for the bulk microscale visualization of low-contrast laser-induced structural modifications of nitrogen centers. In this line, ultrashort-pulse-laser-induced aggregation was recently demonstrated in a high-pressure, high-temperature diamond owing to vacancy- and nickel-impurity-mediated reactions [13].

In this study, we report on PL studies on pulse-energy- and exposure-dependent nanoscale transformations of highly aggregated nitrogen centers into lowly aggregated nitrogen centers in natural IaA+B diamonds photoexcited by sub-picosecond visible-range laser pulses, involving vacancy attachment and vacancy-driven splitting processes.

## 2. Materials and Methods

**Figure 1:**
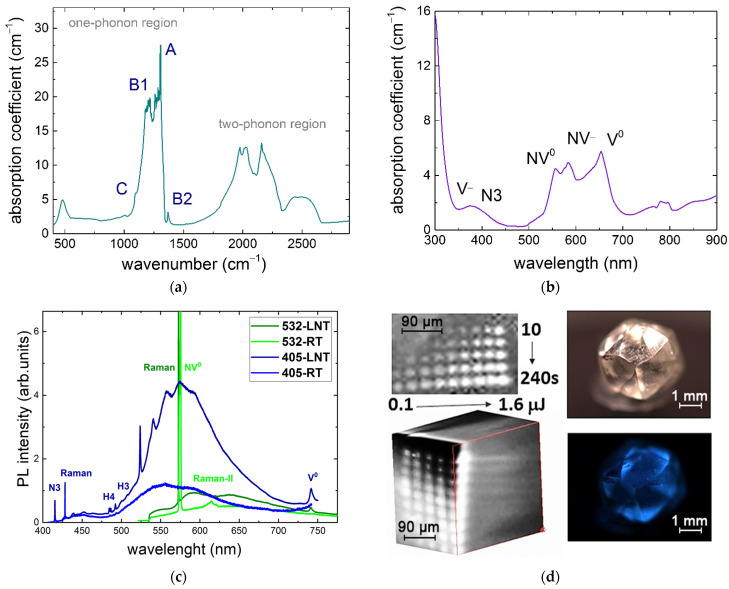
Spectral characterization of the initial diamond sample: (**a**) FT-IR spectrum (spectral assignment after [5]); (**b**) UV–NIR transmission spectrum; (**c**) RT and LNT PL spectra at 405 nm and 532 nm laser excitation; and (**d**) 3D confocal PL-based image of the bulk array of laser-inscribed micromarks (top and side views) as well as PL “Diamond view” images of the diamond.

The experimental sample was a greenish natural high-nitrogen IaA+B diamond crystal (4 × 4 × 4 mm^3^), possessing the average impurity concentrations of [A(2N)]~600 ppm, [B1(4NV)]~90 ppm, some [B2] (platelets), and very minor [3NVH] and negligible [C(N)] abundances according to FT-IR measurements (Figure 1a) obtained using a Vertex V–70 (Bruker, Billerica, MA, USA) spectrometer. Owing to its low intrinsic radioactivity of 6–7 μRg/h above the natural level, its exposed upper layer contained a significant concentration of neutral vacancies, V^0^ (see the PL spectra below). UV–near-IR optical transmission spectroscopy (SF–2000, OKB Spektr, Russia) indicates its green–red (500–700 nm) and UV (<400 nm) absorption (Figure 1b), which could be related to different contributions of N3(3N), NV^0−^, NV^−^, V^0^, and V^−^ centers [5]. These spectral observations are supported by room temperature (25 °C, RT) and liquid nitrogen cooling temperature (−120 °C, LNT) 3D scanning confocal Raman/PL microspectroscopy (inVia InSpect, Renishaw, UK, NA = 0.5, acquisition spot size ≈ 1.5–2 μm) of the buried PL micromarks at laser excitations of 405 nm and 532 nm. In the first case, PL spectra exhibited an N3(3N) band (ZPL at 415 nm), a 1332 cm^−1^ Raman band of optical phonons in the diamond (428 nm), and a broad band of H4(4N2V) centers in the range of 480 to 550 nm (Figure 1c). In the second case, characteristic Raman (573 nm), NV^0^ with its ZPL at 575 nm, NV^−^ with its ZPL at 637 nm, and V^0^ (GR1) at 740 nm bands were excited by a 532 nm laser.

Laser exposure was performed, using a laser workstation and exposure scheme described elsewhere [4,6,8,13,14,15], in a number of patterns by a series of 515 nm, 300 fs pulses, focused by a 0.25-NA micro-objective into the diamond into the focal spot of a 2-μm 1/e-intensity radius at the arbitrary linear focusing (no filamentation accounted for) depth z~360 μm and coming at a 100 kHz repetition rate for 10, 30, 60, 120, and 240 s at variable pulse energies of 0.1, 0.2, 0.3, 0.4, 0.6, 0.8, 1.0, 1.2, 1.4, and 1.6 μJ, making an array of the PL micromarks that range in their visible diameter from 3 to 15 μm (Figure 1d).

## 3. Experimental Results

### 3.1. PL Spectra at 532 nm Laser Photoexcitation

**Figure 2:**
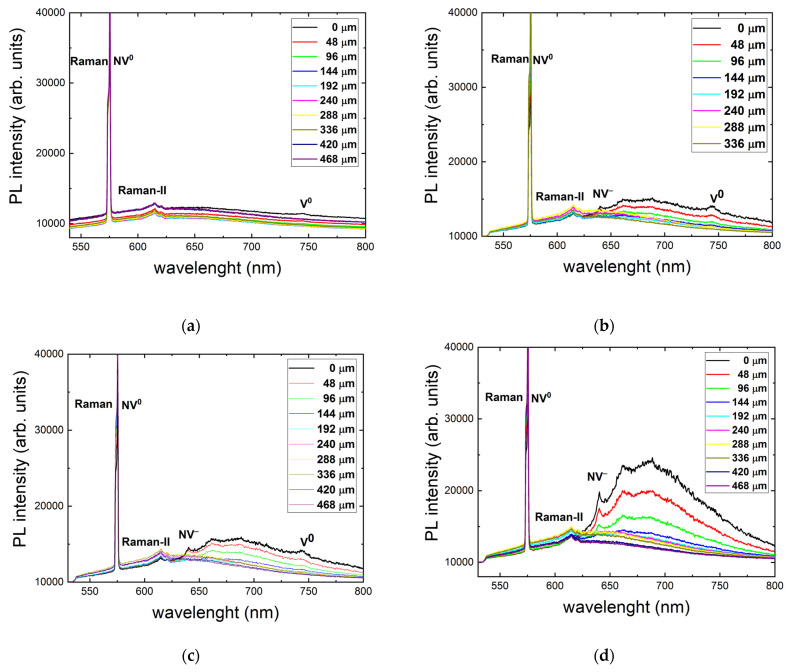

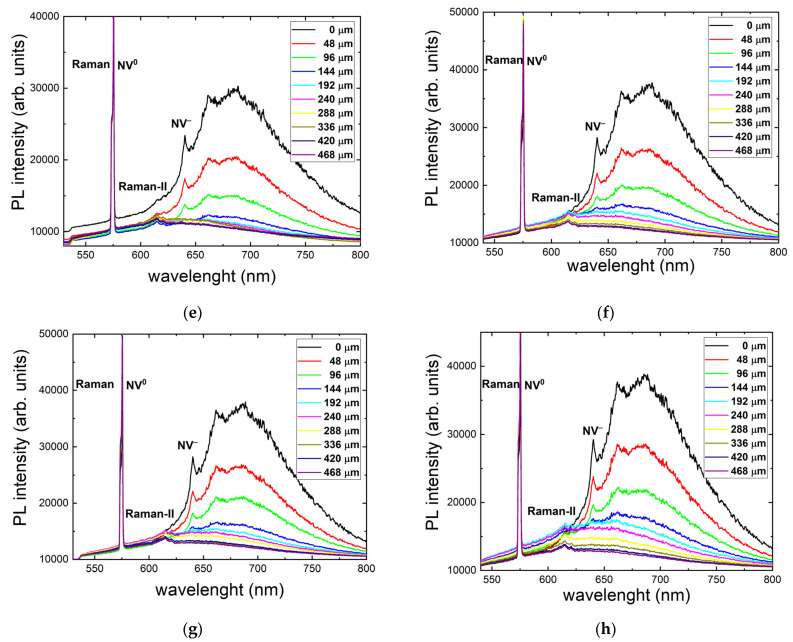
RT 532 nm excitation PL spectra at different depths in μm inside the sample (frames) for an exposure time of 240 s (24 M pulses) and different incident 0.3 ps laser pulse energies (μJ): 0/background (**a**), 0.4 (**b**), 0.6 (**c**), 0.8 (**d**), 1.0 (**e**), 1.2 (**f**), 1.4 (**g**), and 1.6 (**h**). Spectral assignment after [5].

Three-dimensional scanning confocal RT PL microspectroscopy at 532 nm in the unexposed diamond region indicates a weak structureless composition of NV^0^ and NV^−^ bands (Figure 2a) with almost constant intensity regardless of the probing depth of 0–480 μm inside the sample. This indicates that, at least, the sample is rather homogeneous in this spatial region regarding the optical center probed in the PL spectral range, and the microspectroscopy provides truly confocal probing without artefacts of considerable sample absorption of the 532 nm CW laser pump or PL emissions.

**Figure 3:**
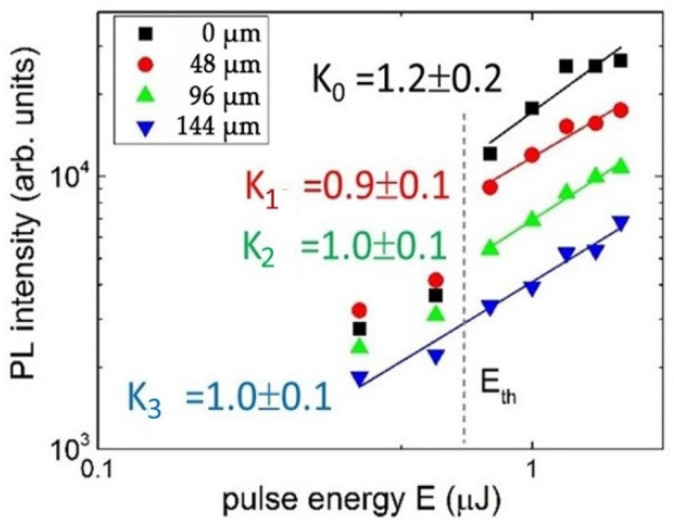
Peak intensity of the NV^−^ band at 685 nm at different depths versus incident 0.3 ps laser pulse energy in log–log coordinates as well as the linear fitting lines of these curves with their slopes, K_0_ (depth—0 μm), K_1_ (depth—48 μm), K_2_ (depth—96 μm) and K_3_ (depth—144 μm), and the threshold energy, E_th_.

Other RT PL spectra in Figure 2 demonstrate the eventual increase in NV center intensity deeper and deeper inside the sample as a function of the 515 nm, 0.3 ps laser pulse energy, ranging from 0.4 to 1.6 μJ. Accounting for the absence of such an effect at the almost same 532 nm wavelength of the PL pump, the observed limited depth for the fs-laser-induced appearance of NV^−^ centers is apparently related to non-linear absorption in the material (coefficient of ~10^3^ cm^−1^), in agreement with the strong absorption edge near 400 nm in Figure 1b. This finding is supported by the linear energy dependence of NV^−^ intensity taken at the maximum of ≈685 nm (Figure 3), where the linear PL excitation regime could result from strong intraband electron–hole plasma absorption and related laser filamentation in diamonds [15], pronounced in the highly elongated shape of the PL micromarks.

**Figure 4:**
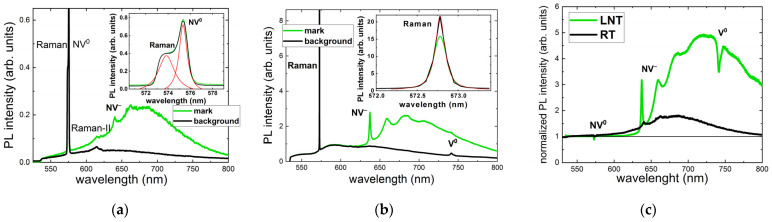
RT (**a**) and LNT (**b**) 532 nm PL spectra of the background, micromarks, and their normalized intensity (**c**) for an exposure time of 240 s (24 M pulses) and an incident 0.3 ps laser pulse energy of 1.6 μJ. Spectral assignment after [5].

More detailed analyses of RT and LNT PL spectra acquired at a depth of 100 μm for a pulse energy of 1.6 μJ and an exposure time of 240 s (24 M pulses) show no NV^0^ ZPL and very minor intensity in the related spectral region of >575 nm in both the background and micromark PL spectra at LNT (Figure 4). In contrast, RT PL spectra indicate the presence of NV^0^ ZPL in both the background and micromark emissions. Moreover, NV^−^ ZPL appears only in the RT and LNT PL spectra of the micromarks, but not for the background. Finally, the V^0^ peak remains only under LNT PL acquisition in both the background and micromark emissions. These data indicate some RT lability of vacancies, the possible RT ionization of NV^−^ centers to NV^0^ ones, and the absence of NV^−^ centers in the background at both RT and LNT.

### 3.2. PL Spectra at 405 nm Photoexcitation

**Figure 5:**
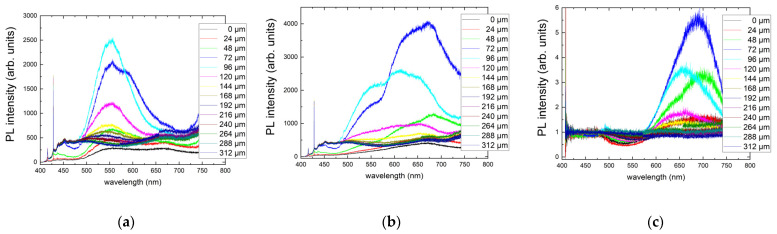
RT 405 nm excitation PL spectra of the background (**a**), micromarks (**b**), and their normalized intensity (**c**) at different depths inside the sample (frames) for an exposure time of 240 s (24 M pulses) and an incident 0.3 ps laser pulse energy of 1.6 μJ. Spectral assignment after [5].

Compared to 3D scanning confocal RT PL microspectroscopy at 532 nm excitation, 405 nm RT PL excitation indicates dramatic spatial and spectral variation in the background PL yield (Figure 5a), apparently in agreement with the UV–Vis absorption spectrum of the sample (Figure 1b) and the potential spatial variation in optical center distribution inside the material. Specifically, the overall rise in PL intensity occurs for the near-surface layer of 50–70 μm thickness, while its green–red part of the PL spectra strongly increases at a depth of 50–100 μm. Similarly, in the PL micromarks the strongest spectral variations in the range of 500 to 700 nm occur at the same depths of 50–100 μm (Figure 5b). In both of these cases, the strong PL yield variations took place in the colored green top layer of the diamond, absorbing the red and UV ranges. For simplicity, a normalization procedure was undertaken to minimize the spatial background effects in analysis of color-center modification inside the micromarks (Figure 5c). Here, the normalized spectra exhibit a systematic near-surface drop in PL intensity in the range of 485 to 575 nm and a simultaneous increase in the range of 575 to 750 nm, representing, apparently, H3, H4, NV^0^, and NV^−^ nitrogen centers [5].

**Figure 6:**
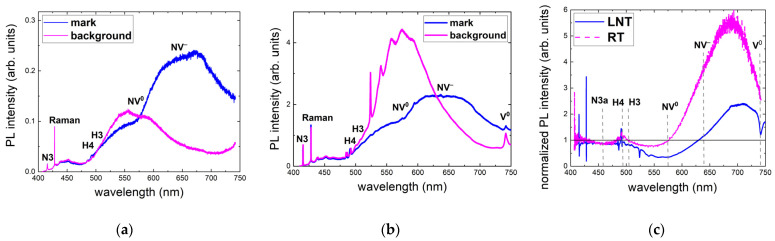
RT (**a**) and LNT (**b**) 405 nm PL spectra of the background and micromarks, as well as their normalized intensity (**c**) for an exposure time of 240 s (24 M pulses) and an incident 0.3 ps laser pulse energy of 1.6 μJ. Spectral assignment after [5].

In support of these observations, RT and LNT 405 nm PL spectra of the background, micromarks, and their normalized intensity were comparatively analyzed at a fixed sub-surface depth of 70 μm (Figure 6), where the spatial PL yield variation is very pronounced (Figure 5). In the RT PL spectra one can see a reduced intensity in the range of 500 to 575 nm at the expense of its following rise (Figure 6a). In contrast, in the LNT PL spectra one can find a reduced intensity in the range of 494 to 630 nm accompanied by a following rise, more consistent with the fs-laser-induced decomposition of H4 centers into NV^−^ ones (Figure 6b), in agreement with our results of the 532 nm PL photoexcitation presented above in Figure 4. Finally, the normalized (micromarks/background) intensity spectra indicate a general decrease in N3 and N3a bands (<500 nm) [5], while at RT the following drop occurs in the range of 500 to 575 nm in comparison to the LNT-acquired drop in the range of 500 to 630 nm. The following rise above 575 nm (RT) or 630 nm (LNT) implies the increased concentration of NV^0^ and NV^−^ centers, respectively, with their ratio dictated by the temperature.

**Figure 7:**
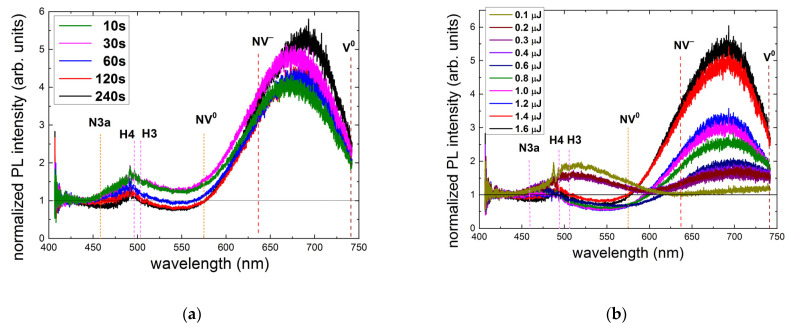
Normalized RT 405 nm PL intensity spectra acquired at a depth of 20 μm at different laser exposures of 1–24 M pulses ((**a**) pulse energy—1.6 μJ) and energies in the range of 0.1 to 1.6 μJ ((**b**) an exposure time of 240 s—24 M pulses). Spectral assignment after [5].

More detailed RT 405 nm confocal PL excitation microspectroscopic studies in terms of laser exposure time and pulse energy uncover the underlying microscopic structural dynamics of the nitrogen center abundance (Figure 7). First, in the exposure-dependent PL spectra at the maximal pulse energy of 1.6 μJ (Figure 7a), one can observe the general initial (10–60 s, 1–6 M pulses) PL yield increase for N3a- (450–550 nm), H3, and H4 centers (496–550 nm), turning to their decrease for the longer exposures (120 and 240 s, 12–24 M pulses). In contrast, the normalized intensity for the NV^0^ center increases drastically versus exposure almost to its saturation.

More interestingly, the pulse-energy-dependent RT 405 nm PL spectra of the normalized intensity exhibit, at low pulse energies (E < 0.6 μJ), a simultaneous rise in the entire PL spectra—both the 445–600 nm and 600–750 nm PL bands, while at the minimal energy of 0.1 μJ—even without a rise in the 630–750 nm band (Figure 7b). This finding indicates the initial low-energy yield of N3a, H3, and H4 centers, which only start to transform into NV centers at higher energies. Surprisingly, the threshold energy of 0.6 μJ for this transition from the rising 445–600 nm band to the dropped one is the same as E_th_ in Figure 3 for the threshold rise in the PL intensity of NV centers. These features of laser exposure and energy are discussed in the next section.

## 4. Discussion

Although the 532 nm PL studies only exhibit a general rise in the NV center in the nanostructured micromarks, 405 nm PL spectral acquisition indicates much more complex structural transformations of nitrogen centers, involving N3a, H3, and H4 ones. Indeed, N3 centers are visibly present in Figure 6 and Figure 7 in the unexposed material, while their interaction with the present B2 centers (platelets [5]) results in red-shifted PL spectral appearances of N3a centers (ZPL at 461 nm, mostly at liquid helium temperatures in natural diamonds [5,16,17,18,19]).

Other modifications of the present A, N3, B1, and B2 centers could be assigned to the following low-energy aggregative, high-energy dissociative, and vacancy-driven splitting transformations:

(1) $$\begin{array}{l} \overset{}{\mathrm{Vacancy}-\mathrm{driven}\;\mathrm{aggregation}}\\ C_{S}\to C_{I}+V;\\ 2N(A)+V\to 2\mathrm{NV}(H3);\\ 4\mathrm{NV}(B1)+V\to 4N2V(H4);\\ 2N(A)+\mathrm{NV}\to 3\mathrm{NV}(N3).\\ \overset{}{\mathrm{High}-\mathrm{energy}\;\mathrm{dissociation}}\\ 4\mathrm{NV}(B1)\to 2N(A)+2\mathrm{NV}(H3);\\ 4N2V(H4)\to 3\mathrm{NV}(N3)+\mathrm{NV};\\ 2\mathrm{NV}(H3)\to 2N(A)+V;\\ 2\mathrm{NV}(H3)\to N(C)+\mathrm{NV}\\ 2N(A)\to N(C)+N(C);\;N(C)+V\to \mathrm{NV}.\\ \overset{}{\mathrm{Vacancy}-\mathrm{driven}\;\mathrm{splitting}}\\ 2N(A)+V+V\to \mathrm{NV}+\mathrm{NV};\\ 2\mathrm{NV}(H3)+V\to \mathrm{NV}+\mathrm{NV};\\ 4\mathrm{NV}(B1)+V+V+V\to \mathrm{NV}+\ldots .+\mathrm{NV};\\ 4N2V(H4)+V+V\to \mathrm{NV}+\ldots .+\mathrm{NV}. \end{array}$$

Here, according to our experimental data in Section 3.2, low-energy fs laser excitation promotes the aggregation of nitrogen centers till N3, H3, and H4, involving vacancies at low concentrations, similarly to their aggregation at moderate temperatures ≈1800–2000 °C [20]. Simultaneously, N3a (N3–B2 aggregates) centers appear at a higher concentration at these conditions due to the enhanced yield of N3 centers, both free and interacting with B2 centers.

In contrast, at higher fs laser pulse energies, similarly to high-temperature treatments (>2300 °C [20]), the dissociation of the highly aggregated nitrogen centers A, H3, H4, and B2, as well as interactions with vacancies, proceeds in favor of lowly aggregated ones (NV, …). In this regime, the vacancy-driven splitting of the highly aggregated nitrogen centers H3, H4, and B2 could also occur in a concerted way at high vacancy concentrations. Specifically, the naturally present, intrinsic vacancies, V^0^, in the weakly radioactive diamond disappear in this high fs laser energy regime of exposure, being consumed for the latter type of reactions.

## 5. Conclusions

In conclusion, multi-pulse 0.3 ps, 515 nm laser micro-inscription inside a bulk natural IaA+B diamond of a bulk array of photoluminescent nanostructured microtracks as a function of pulse energy and exposure indicated the low-energy and exposure rise in vacancy-enriched nitrogen centers, such as N3a, H3, and H4. In contrast, at higher, above-threshold pulse energies, the laser-induced dissociation of highly aggregated nitrogen centers in the vacancy-enriched focal volume resulted in predominating NV centers. The concerted splitting of the highly aggregated nitrogen centers surrounded by vacancies at high concentrations occurred until NV centers could also become possible.

## Data Availability

The data supporting the reported results are accompanying this submission.
